# Visualisation of in vivo protein synthesis during mycobacterial infection through [^68^Ga]Ga-DOTA-puromycin µPET/MRI

**DOI:** 10.1038/s41598-024-70200-4

**Published:** 2024-08-20

**Authors:** Sebastian Eigner, Janke Kleynhans, Dennis R. Beckford Vera, Mike M. Sathekge, Katerina Eigner Henke, Thomas Ebenhan

**Affiliations:** 1 Department of Radiopharmaceuticals, Nuclear Physics Institute of the Academy of Sciences of the Czech Republic, Husinec-Rez 130, 25068 Husinec-Rez, Czech Republic; 2 Department of Radiopharmacy, Charles University Prague, 11000 Prague, Czech Republic; 3 Nuclear Medicine Research Infrastructure NPC, Pretoria, 0001 South Africa; 4 Department of Nuclear Medicine, University of Pretoria, Pretoria, 0001 South Africa; 5 Clinical for Nuclear Medicine, University Hospital RWTH Aachen, 52074 Aachen, Germany

**Keywords:** Mycobacterium tuberculosis, Positron emission tomography/magnetic resonance imaging, Fluorodeoxyglucose, Gallium-68, Molecular imaging, Molecular medicine, Medical research

## Abstract

Radiolabelled puromycin analogues will allow the quantification of protein synthesis through nuclear medicine-based imaging. A particularly useful application could be the non-invasive longitudinal visualisation of mycobacterial activity through direct quantification of puromycin binding. This study assesses the value of [^68^Ga]Ga-DOTA-puromycin in the visualisation of mycobacteria through positron emission tomography combined with magnetic resonance imaging (µPET/MRI). The radiopharmaceutical was produced by previously published and validated methods. [^68^Ga]Ga-DOTA-Puromycin imaging was performed on severe immunodeficient mice infected with Bacille Calmette-Guérin-derived *M. Bovis* (BCG). Acute and chronic infection stages were examined by µPET/MRI. A follow-up group of animals acted as controls (animals bearing *S*. *aureus*-derived infection and sterile inflammation) to assess tracer selectivity. [^68^Ga]Ga-DOTA-puromycin-µPET/MRI images revealed the acute, widespread infection within the right upper shoulder and armpit. Also, [^68^Ga]Ga-DOTA-puromycin signal sensitivity measured after a 12-week period was lower than that of [^18^F]FDG-PET in the same animals. A suitable correlation between normalised uptake values (NUV) and gold standard histopathological analysis confirms accurate tracer accumulation in viable bacteria. The radiopharmaceutical showed infection selectivity over inflammation but accumulated in both *M. Bovis* and *S. Aureus*, lacking pathogen specificity. Overall, [^68^Ga]Ga-DOTA-puromycin exhibits potential as a tool for non-invasive protein synthesis visualization, albeit without pathogen selectivity.

## Introduction

In the field of nuclear medicine, the enhancement of nuclear imaging techniques for infections is a prominent area of focus. Existing radiopharmaceuticals like [^67/68^Ga]Ga-citrate and [^18^F]Fluorodeoxyglucose ([18F]FDG) are capable of visualizing broad inflammatory responses, but they do not enable specific visualization of the underlying microorganism-associated pathology^1,2^. The distinction between infection and inflammation is a critical medical need as this can result in completely different clinical strategies followed in patient care^3^. Other co-morbidities can further influence the specificity of current radiopharmaceuticals^4,5^. It has indeed been demonstrated that [^18^F]FDG accumulates in sites of inflammation caused by the human immunodeficiency virus (HIV)^6^. This phenomenon severely hampers the ability of these tracers to identify tuberculosis (TB) lesions in HIV positive patients. Since global data in 2018 indicated that 10 million people were diagnosed with TB and 1.5 million related deaths occurred worldwide, an early diagnosis and staging thereof is of utmost importance^7^.

Puromycin is known as a compound with antibiotic activity that targets both prokaryotic and eukaryotic cells and acts as a universal protein synthesis inhibitor. It is structurally related to the aminoacyl-moiety of the aminoacyl-tRNA and binds competitively to the ribosomal A-site. It is believed that the binding of puromycin is by covalent attachment to the nascent peptide chain during protein synthesis^8–11^. It ultimately blocks the incorporation of the next aminoacyl-tRNA and therefore terminates protein synthesis. Puromycin labelled with biotin, as a molecular tool in biology, has been extensively utilized to reveal alterations of protein synthesis processes in vivo. The biotin moiety demonstrates exhibits analogous traits to the bifunctional chelator 1,4,7,10-teraazyclododecane-1,4,7,10-tetraacetic acid (DOTA) and this led to the development of radiometal-labelled puromycin tracers for potential applications in nuclear medicine imaging^12^ for the detection of cellular ribosomal activity. Puromycin derivatives have been labelled with fluorine-18, carbon-11 and scandium-44 previously^12–15^. Successful imaging of protein synthesis in vivo to visualise cancer in a xenograft model (AT1 cells in male Copenhagen rats) was previously reported^12^.

Compared to other radiometals, the advantages of applications of gallium-68 radiopharmaceuticals have been reviewed extensively^16^, attributed to convenience of ^68^Ge/^68^Ga generators and improved imaging properties relative to SPECT imaging. The successful labelling of DOTA-puromycin with gallium-68, a PET (Positron Emission Tomography) radionuclide with a 68-min half-life, was previously reported^12^. Although radiolabelling of infection targeting ligands with technetium-99m (such as antibiotics) is a prolific area of research^17^, infection imaging agents incorporating gallium-68 would be more optimal owing to the improved spatial resolution of PET cameras compared to that of technetium-99m SPECT (Single-Photon Emission Computed Tomography) radiopharmaceuticals^16^.

To date, no systematic characterization of a puromycin-based radiopharmaceutical as a strategy for nuclear medical infection imaging has been reported. The authors gained preliminary knowledge on the potential value and role of puromycin-based PET-imaging by performing a small pilot study in infected rabbits^18^. The results stimulated the narrative that targeting bacterial infection at the level of protein synthesis could be a valuable approach for visualising the affected tissue noninvasively. This study aimed to assess the specificity of a non-invasive nuclear imaging technique (µPET/MRI) utilizing [^68^Ga]Ga-DOTA-puromycin. The goal was to ascertain its potential as a valuable method for visualizing mycobacterial infections.

## Results

### Radiolabelling and tracer administration

DOTA-puromycin was effectively labelled with gallium-68 with a total synthesis duration of 60 min. The ensuing purified formulation was buffered with PBS at physiological pH and demonstrated a radiochemical purity suitable for injection 97.0 ± 1.1% (N = 5). Radiosynthesis yields amounted to 58.0 ± 7.0 MBq (62–68%; n > 3). Mice received [^68^Ga]Ga-DOTA-puromycin 0.31–0.39 MBq/g (total dose of 7.6 ± 1.6 MBq) and the specific activity was calculated to be 1.5 ± 0.1 GBq/µmol. All injections were administered as a 0.13 ml intravenous bolus (11.4 ± 2.4 nmol DOTA-puromycin/ mouse). The [^18^F]FDG was administered as a 0.10–0.13 ml intravenous bolus containing 8.8 ± 0.7 MBq tracer.

### Animal response to infection

The study effectively achieved its goals by investigating two distinct objectives. The first objective centred around tracer imaging accuracy and specificity, while the second objective focused on tracer selectivity. These investigations were conducted using two separate animal groups. Prior pilot tests were performed during the development of the animal model to determine the inoculated BCG dose needed to result in pathology of infection and successful infection was evaluated through the presence of sufficient levels of acid-fast-bacilli during staining of infected tissue ex vivo. For group 1 (N = 20) in this study, 6 mice were used after 12 weeks to confirm sufficient bacterial load in the infected lungs and percutaneous tissues. Humane endpoint euthanasia was performed in 4/20 (20%) animals due to inacceptable severity of symptoms. The remaining set of animals (n = 10) showed acceptable symptoms and fitness to be used for the acute and chronic disease state groups. Those animals tolerated the acute interventions well. Subsequently the state of disease progression was acceptable to study long-term chronic effects of BCG. All 5 animals of group 2 showed acceptable symptoms and fitness to perform the required imaging procedures.

### [^68^Ga]Ga-DOTA-puromycin-µPET/MRI imaging of acute BCG infection

**Figure 1:**
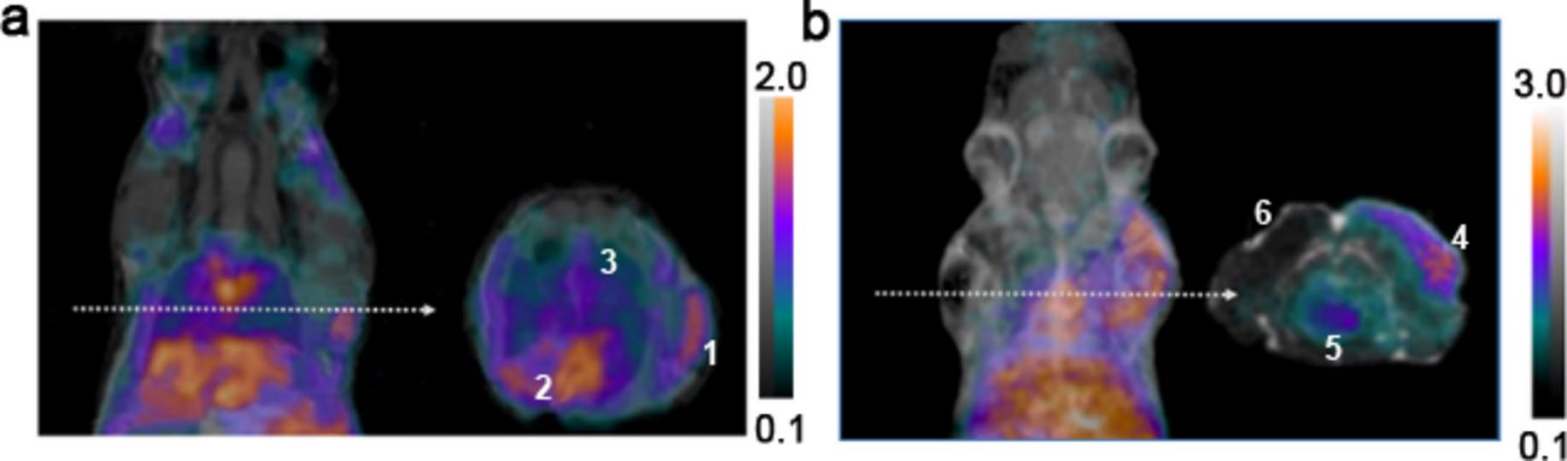
(**a**) [^68^ Ga]Ga-DOTA-puromycin-µPET/MRI imaging for detection of acute BCG infection. Representative coronal and axial µPET/MRI image slides (white arrows indicate the special areal of axial slides) of a BCG-infected SCID mouse acquired at 45 min post tracer injection (1) percutaneous BCG-infection site (right front leg); (2) myocardium; (3) pulmonary BCG-derived infection. (**b**) [^68^ Ga]Ga-DOTA-puromycin-µPET/MRI imaging testing for tracer selectivity. A maximum image projection and a representative slide in axial orientation (arrow) are displayed following injection of 6.9 MBq tracer; (4) *S*. *aureus*-derived infection in the right shoulder area; (5) myocardium; 6) injection site for sterile turpentine oil (left shoulder area).

Animals from group 1 received a percutaneous (right front leg) BCG dose and [^68^Ga]Ga-DOTA-puromycin-µPET/MRI imaging was performed at 24 h after inoculation. All µPET/MRI images (n = 5) clearly indicated elevated, dispersed radioactivity as displayed at the injection site of the right front leg (Fig. 1a**, **#1). The presence of activity in the lower bronchial tract (Fig. 1a**, **#3) may be attributed to the acute state after inhalation of BCG.

Results from the image-guided tissue quantification are summarised in Table 1 and correlated with the microanatomical changes as indicators of inflammation and bacilli-specific Ziehl–Neelsen staining. A normalised uptake value (NUV) of 0.69 ± 0.16 was observed for [^68^Ga]Ga-DOTA-puromycin in the percutaneous infection site. No tracer uptake was seen in the sham inoculation area (saline) which allowed for a calculated target to non-target ratio of 2.79 ± 0.45 representing the BCG inoculation site. The BCG-derived pulmonary infection site (low density) had much lower NUV and T/NT ratios. Both NUV and T/NT ratios correlated well with the histology gradings presented as these also indicated a lower rate of infection in the pulmonary tissue compared to the inoculated area.

**Table 1:** Acute BCG-Infection—[^68^ Ga]Ga-DOTA-puromycin Image-guided Quantification and ex vivo Tissue Histopathology.

Tissue/organ	[^68^ Ga]Ga-DOTA-puromycin imaging			Histology (grading)	
	NUV	T/NT^¥^	T/Ref*	ZN	H&E
BCG (pcn.)	0.69 ± 0.16	2.79 ± 0.45	3.80 ± 0.66	3.38 ± 1.39	2.21 ± 1.18
BCG (pulm.)^**†**^	0.28 ± 0.09	1.39 ± 0.31	1.37 ± 0.19	2.15 ± 0.44	2.05 ± 0.45
Myocardium	0.78 ± 0.34	–	4.22 ± 1.25	Nd	1.15 ± 0.15
Liver	0.98 ± 0.64	–	5.05 ± 1.83	Nd	1.45 ± 0.90
Spleen	0.33 ± 0.13	–	1.95 ± 0.71	Nd	1.26 ± 0.27

Results are expressed as mean values (± SD; N ≥ 5; ♀/♂). *Ref,  reference tissue (details see methods), e.g., radioactivity represented within a cubic volume-of interest area was used. ^¥^T/NT, target-to-non-targeted ratio; non-targeted tissue chosen as following: left armpit (e.g., negative control area injected with sterile saline solution) and intrascapular neck/scruff area (for BCG pulm.). ^†^Pulm., pulmonary, i.e., whole lung volume-of-interest was used to calculate NUV. Nd, no detection of acid-fast bacilli; Pcn., percutaneous infection site (right front leg). ZN, Ziehl–Neelsen; H&E, Hemotoxin & Eosin.

A parallel investigation (Group 2) was performed as a control to determine tracer selectivity for mycobacteria species above sterile inflammation or gram-positive infections (i.e., *S. aureus*-derived subcutaneous infection). Animals from group 2, showed clear uptake in the *S. Aureus* inoculation site (Fig. 1b, #4), but negligible signal for the contralateral sterile inflammation site (Fig. 1b, #6).

Results from the image-guided tissue quantification are summarised in Table 2 and correlated with the microanatomical changes as indicators of inflammation and Ziehl–Neelsen staining. At the inflammation site no noteworthy accumulation of [^68^Ga]Ga-DOTA-puromycin was present. The NUV of the *S. Aureus* inoculation area was 0.78 ± 0.15 with a target to non-target ratio of 3.57 ± 0.29. This is not significantly different when compared to the BCG inoculation site indicating that the tracer is non-selective towards certain types of infections (i.e., a TB specific radiopharmaceutical). Additional ex-vivo biodistribution data and images are also included in the Supplementary Figure S3 and S4.

**Table 2:** Tracer Selectivity for [^68^Ga]Ga-DOTA-puromycin-PET/MRI Image-guided Quantification and Histology.

Tissue type	[^68^Ga]Ga-DOTA-puromycin imaging			Tissue Pathology (grading)	
	NUV	T/NT^¥^	T/Ref*	Gram +	H&E
SA (sc. infection)	0.78 ± 0.15	3.57 ± 0.29	4.88 ± 0.63	4.25 ± 1.13	1.85 ± 0.74
STO (inflammation)	0.17 ± 0.11	1.10 ± 0.04	1.35 ± 0.19	Nd	2.45 ± 0.39

^**†**^Results are expressed as mean values (± SD; N = 3–5; ♀/♂). *Ref, reference tissue (see details in methods), e.g., radioactivity represented within a cubic volume-of interest area was used. ^¥^T/NT, target-to-non-targeted ratio : signal from muscle tissue of both hind legs. Nd, no detection of bacteria. sc., subcutaneous; SA, Staphylococcus aureus injection site; STO, sterile turpentine oil injection site; Gram+ , tissue staining for gram-positive bacteria; H&E Haematoxylin and eosin tissue stain.

### Non-invasive monitoring of chronic BCG infection

**Figure 2:**
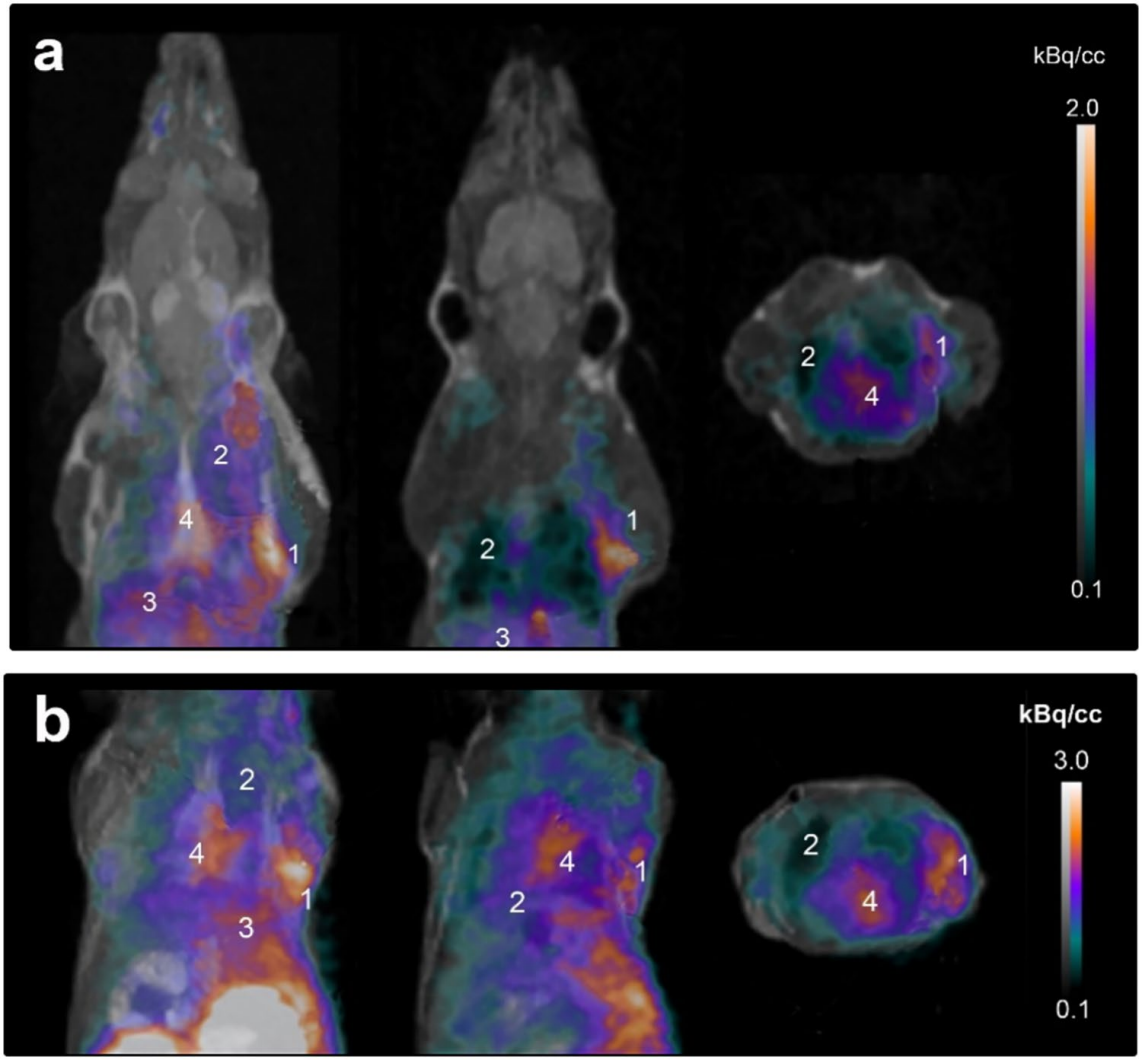
Noninvasive µPET/MRI Imaging chronic BCG infection. (**a**) [^68^ Ga]Ga-DOTA-puromycin-µPET/MRI images, (**b**) [^18^F]FDG-µPET/MRI images. Representative imaging of infected SCID mice 12 weeks after percutaneous inoculation (right front leg/armpit) and intranasal droplet dissemination of BCG into lung tissue in (left to right) maximum intensity projection (left), coronal (middle) and axial (right) orientation. For **a** and **b** numbers reflect tissue as follows: (1) percutaneous infection site, (2) healthy lung areal, (3) liver, and (4) myocardium.

The µPET/MRI images acquired 12 weeks after BCG administration (Fig. 2) clearly visualised widespread infection (in both modalities) on the right upper torso originating from the infection induced in the armpit aerial. Low density BCG foci in the lung (2nd infection site) could not be clearly identified, instead a diffuse, slightly elevated tissue signal is evident. High radioactivity in kidneys and bladder confirmed prior findings on the main excretion pathway via a renal clearance.

The µPET/MRI image guided analysis of relevant tissues (Table 3) revealed high variabilities for both [^18^F]FDG and [^68^Ga]Ga-DOTA-Puromycin NUVs. [^18^F]FDG NUVs were much higher for infected tissues (sevenfold for percutaneous tissue and a 13-fold for pulmonary tissue) over the healthy tissue references and twofold higher than the hepatic tracer concentration. In contrast, [^68^Ga]Ga-DOTA-puromycin NUVs were moderate to low, however a fourfold higher percutaneous tissue concentration and a twofold for pulmonary tracer concentration was calculated over the healthy tissue references. The liver NUV range for [^68^Ga]Ga-DOTA-puromycin (0.12 – 0.21) was favourably lower compared to [^18^F]FDG (0.33–0.42). The total hepatic tracer uptake was 3.51 ± 0.39%ID/g.

**Table 3:** Head-to-head comparison of tracer tissue concentrations and pathohistological quantification.

Tissue/Organ	Tissue concentration (NUV)*		Tissue pathology (grading)*	
	[^68^ Ga]Ga-DOTA-puromycin	[^18^F]FDG	Infection (ZN staining)	Inflammation (H&E staining)
Healthy (pcn.)	0.08 ± 0.02	0.11 ± 0.05	Nd	Nd
BCG (pcn.)	0.35 ± 0.13	0.79 ± 0.32	4.32 ± 1.12	2.33 ± 0.48
BCG (pulm.)	0.19 ± 0.03	0.72 ± 0.15	5.37 ± 1.82	1.76 ± 0.54
Liver	0.15 ± 0.03	0.38 ± 0.20	Nd	1.05 ± 0.22

*Results are expressed as mean values (± SD; N = 3–5; ♀/♂). Pcn., percutaneous infection site (right front leg); pulm., pulmonary BCG infection site; ZN, Ziehl–Neelsen; H&E, Hemotoxin & Eosin; Nd, no detection of Gram-positive/acid-fast bacteria.

Evidence of mycobacterial manifestation was confirmed by tissue staining with grade 4 in the percutaneous BCG infection site and grade 5 for the infected lungs (not whole lungs). Evidence of systemic mycobacterial disease was suggested by change in animal behaviour but was not confirmed by the data—no inflammation in spleen (not shown) and liver.

**Figure 3:**
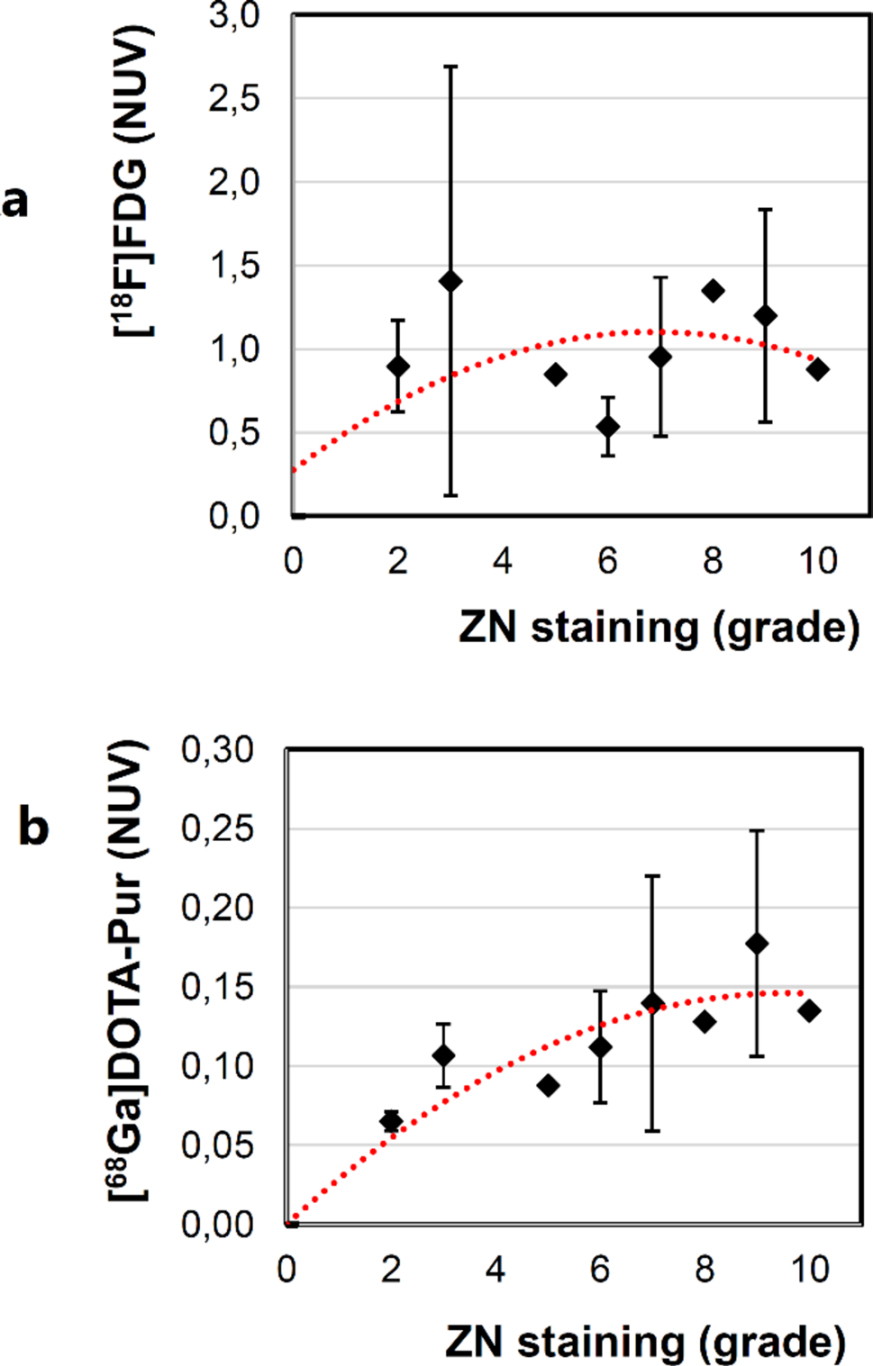
Spearman-rank order correlation plot for NUV values and ZN staining grade (N = 16) measuring. Power regression (r^2^) analysis returned values of (**a**) 0.390 and (**b**) 0.855. (**a**) correlation between ZN staining grade versus [^18^F]FDG NUV (rho = 0190; p = 0.476; S = 549), (**b**) correlation between ZN staining grade versus r [^68^ Ga]Ga-DOTA-puromycin NUV (rho = 0.735; *p* = 0.0012; S = 180) in all infectious foci. Between a and b factor 10 was applied to the y-scale to afford optimal display of the results.

In addition, tracer sensitivity for [^68^Ga]Ga-DOTA-puromycin- and [^18^F]FDG-µPET was determined from randomised pulmonary tissue sections from 4 animals (ZN staining and grading of bacterial burden) to be correlated with the tracer concentration (NUV) for each of the lung lobes. On average, the [^68^Ga]Ga-DOTA-puromycin NUV, the [^18^F]FDG NUV and ZN-stain based grading were, 0.12 ± 0.05, 1.02 ± 0.59 and 5.56 ± 2.67, respectively (N = 16). For [^68^Ga]Ga-DOTA-puromycin, only bacterial burden levels 7–10 reflected in sufficiently high NUV to delineate the lung pathology. With all (16/16) [^18^F]FDG NUV values being elevated (range: 0.41 -2.89, also plotted in Fig. 3a), expected lack of tracer sensitivity was confirmed and systemic mycobacterial pneumonia was likely.

For signal accuracy, Spearman's rank correlation test resulted in an adequate correlation of the [^68^Ga]Ga-DOTA-puromycin NUV to the level of mycobacterial presence ZN stain grading (Fig. 3b); but [^18^F]FDG NUV was non-correlative. In addition, a miscorrelation between [^18^F]FDG- and [^68^Ga]Ga-DOTA-puromycin values was observed (Supplementary Figure S1). However, [^18^F]FDG-PET provided approximately tenfold higher NUV values than the [^68^Ga]Ga-DOTA-puromycin (Supplementary Figure S1). This indicates that the gallium-68 based tracer maybe more accurate but its imaging performance cannot outperform [^18^F]FDG-PET for the quantification of pulmonary BCG-derived infection in vivo.

## Discussion

PET imaging has gained global prominence as an increasingly employed technique, recognized for its robust utility in non-invasive clinical disease diagnosis, as well as in monitoring responses and assessing outcomes of drug treatments. PET is advantageous in the fact that it offers quantitative imaging of physiological function. This is of particular importance for smaller regions of interest such as infective foci. Eigner et al*.* have originated the radiolabelling of puromycin with the PET radionuclides scandium-44 and gallium-68 and over the past 10 years both carbon-11- and flourine-18-labeled puromycin derivatives were developed accordingly^13–15^. Such a development may help to provide a platform for clinical trial investigations soon, provided that the role and clinical values of radiolabelled puromycin derivatives are clearly defined.

Some factors need to be considered when using Puromycin as an infection imaging agent namely the presence of puromycin resistant organisms and secondly the non-selectivity of Puromycin for prokaryotic cells. Resistance towards puromycin takes place through enzymatic inhibition of puromycin or through alterations in cell wall permeability. However, resistance towards puromycin is highly unlikely as this is not an antibiotic that is used to treat human infections^19^. More often, it is used as a selection antibiotic for genetically engineered cell-lines or as a probe to evaluate protein synthesis^20^.

Puromycin binds non-selectively binds to ribosomes, mimicking the structure of aminoacyl-tRNA. This allows puromycin to enter the ribosome's A-site, where it is incorporated into the nascent peptide chain. Due to its structural mimicry, puromycin can affect both prokaryotic and eukaryotic ribosomes, though its effectiveness can vary between different organisms. Tissues with high metabolic activity and rapid protein synthesis may also accumulate the puromycin based radiopharmaceutical^20^. In such a case, pitfalls for image evaluation might include the presence of cancer lesions. It is therefore an excellent vector to image protein synthesis^21^ but interpretation of the results will happen within the clinical context.

**Figure 4:**
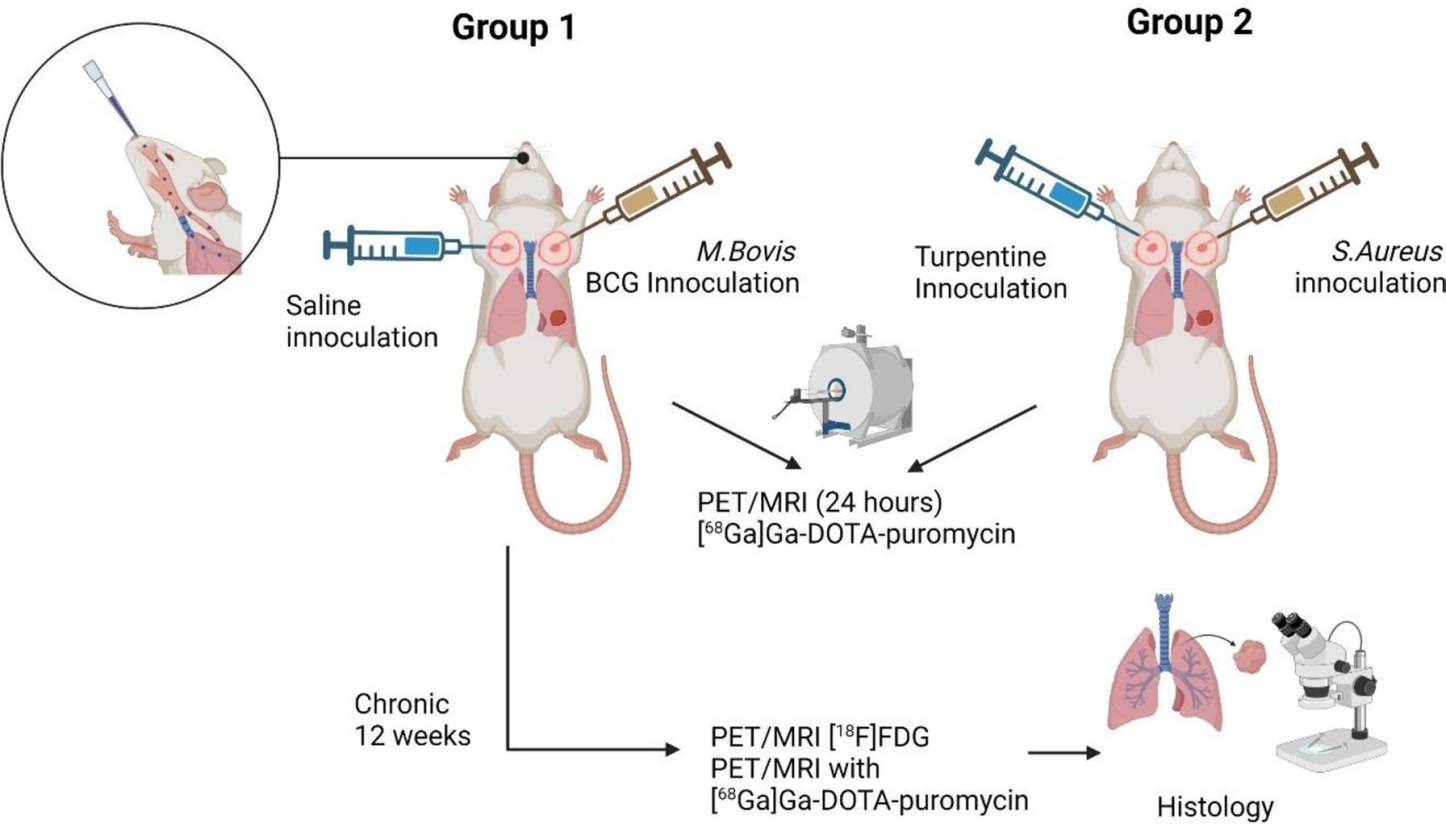
Study design and overview of in vivo/ex vivo experiments (Drawn on BioRender).

When comparing currently readily available PET radionuclides, generator-based gallium-68 would be a practical candidate for potential application in infection imaging. Gallium-68 support features a decentralised production also applicable to less developed hospital radiopharmacies, a frequented elution of radioactivity per day (i.e., radionuclide accessible on-demand), a generator compliance to GMP and GRPP regulations, minimal down-time, and tailored radiosynthesis properties to the tracer’s requirements. It is also a very cost-efficient radionuclide^16^. Due to these advantages, this study was performed with [^68^Ga]Ga-DOTA-puromycin and featured µPET/MRI imaging on infected mice to investigate whether this non-invasive nuclear imaging technique is beneficial to visualise mycobacterial infections in vivo. More details of the study scope are provided in Fig. 4.

Puromycin was discovered as one of the first inhibitors of protein synthesis, and the process of protein translation and the macromolecular ribosome were among the first recognized molecular targets for antibiotics. During protein synthesis both the endogenous and radiolabelled puromycin mimic the aminoacyl-tRNA and thereby bind to the free “A-site” of a ribosome. Catalytic formation of a bond between the nascent polypeptide and puromycin is followed by the release of the peptidyl-puromycin from the ribosome, as no further elongation is possible. The specific effects of puromycin have been used to investigate nascent chain length, the kinetics of chain elongation, and the identification of the effects of other antibiotics^8–11^.

We hereby present the necessary results that prove the performance of [^68^Ga]Ga-DOTA-puromycin-PET/MRI imaging on bacterial ribosomal activity. Firstly, as missing from other literature reports, we achieved a meaningful correlation between the tracer tissue concentration (NUV) and the quantification of the mycobacterial tissue burden (ex vivo) using histopathological analysis (ZN staining). Those results are indicative that the imaging technique accurately in line with the level of infection which allows us to suggest that radiolabelled puromycin indeed accumulates in viable bacteria by the latter described mechanism of action. Some evidence is provided to demonstrate that it could be useful for non-invasive assessment of chronic changes, however this needs further investigation. The accurate representation of the tissue pathology has been reviewed for other antibiotic-based infection imaging agents^22^. Hereby, the advantage of a well understood mechanism of action and a non-compromising radiosynthesis strategy (as applicable to [^68^Ga]Ga-DOTA-puromycin) forms part of the motivation for potential nuclear medicine applications. However, using antibiotic derived radiopharmaceuticals might be challenged by lower imaging sensitivity hinging on the aspect that bacteria might still be exposed to antimicrobial action. Addressing the latter concern, we also provide adequate results that demonstrate lower NUV’s on the [^68^Ga]Ga-DOTA-puromycin scan compared to [^18^F]FDG providing some evidence that it might be less sensitive than the standard [^18^F]FDG scan. This could be detrimental to possible clinical translation for mycobacterium infection specific imaging. However, in this study, the [^68^Ga]Ga-DOTA-puromycin NUV’s calculated during acute the infection phase in mice allowed for clear visualisation of the infection site. The results also support the argument that [^68^Ga]Ga-DOTA-puromycin can play a critical role as a research tool for the preclinical setting—given its limit of detection (lowest detectable tracer concentration over unspecific tissue concentration) is provided, measuring protein synthesis as an in vivo, non-invasive and longitudinal biomarker may provide immense value and significance, for example during drug development or monitoring disease progression. For example, puromycin reactivity using fluorescent or bioluminescent intensity was the limiting factor into study drug action against *C. elegans*^22^. However, the herein described [^68^Ga]Ga-DOTA-puromycin is capable of delivering as little as 1.5 Bq gallium-68 radioactivity per fmol DOTA-puromycin to an in vivo setting, though the study setup was not directed to determine the actual in vivo detection limit for [^68^Ga]Ga-DOTA-puromycin.

Based on the latter finding the tracer was assessed for its imaging selectivity using commonly described control animals. As the tracer accumulated equally well in *S. Aureus* lesions and *M. Bovis* lesions, results provide sufficient evidence to categorize [^68^Ga]Ga-DOTA-puromycin as only selective for infections versus inflammation, but not more selective towards mycobacteria species over other bacteria such as the *S. aureus* tested in this study (pan-bacterial uptake should still be tested in future studies to further confirm selectivity). [^68^Ga]Ga-DOTA-puromycin is therefore not a mycobacterium-specific targeting radiopharmaceutical; however, as the proven tissue response from sterile inflammation was not detectable by [^68^Ga]Ga-DOTA-puromycin-PET/MRI, this technique could be suggested in the research sector for example to test for drug efficacy. For general understanding a representative [^68^Ga]Ga-DOTA-puromycin-µPET/MRI whole-body (neck-pelvis) projection 45 min post injection (Supplementary Figure S4) demonstrates expected tracer distribution in liver, spleen, and predominant renal excretion with higher radioactivity levels found in kidneys and the urinary bladder, which is characteristic for small, polar molecules.

Although a genetically different ribosome complex exists for bacteria, the actual ribosomal function with the protein synthesis and translation as a cellular mechanism is not limited to bacteria^23^. Our previous investigations confirmed [^44^Sc]Sc-DOTA-puromycin uptake in human cancer cell lines and tumour bearing rats^15^. To date, no such explorations in humans have been reported to confirm the value of this nuclear medicine method for imaging cancer.

It is our belief that this radiopharmaceutical in alignment with sensitive PET imaging does have vast applications whenever protein synthesis is investigated as a biomarker. This may include but is not limited to infection imaging but could also be targeting malignancies and neurodegenerative disorders (via reduction)^14^.

## Conclusion

Study results concerning the adequate evaluation of the [^68^Ga]Ga-DOTA-puromycin PET/MRI imaging performance regarding infections derived from in vivo deposition of the BCG agent is presented. The accurate correlation achieved between tracer tissue concentration to gold standard methods in histopathology provides [^68^Ga]Ga-DOTA-puromycin value and potential in preclinical research settings. The low [^68^Ga]Ga-DOTA-puromycin imaging sensitivity during investigations of the chronic disease stage in comparison to [^18^F]FDG-PET may be a limitation for future studies in humans which should be further investigated.

## Methods

### Materials

The 1,4,7,10-tetraazacyclododecane-1,4,7,10-tetraacetic-acid-mono-N-hydroxysuccinimidyl-6-deoxycytidine-puromycin (DOTA-Puromycin) was purchased from Purimex (Grebenstein, Germany) and released with 98% purity for straightforward radiolabelling^12^. Hydrochloric acid, sodium acetate trihydrate (NaOAc) and ethanol were obtained from Sigma-Aldrich (Darmstadt, Germany). Gallium-68 radioactivity was eluted from a commercially available 1.11 GBq ^68^Ge/^68^Ga-generator (ITG, Garching, Germany). Sterile saline (0.9%) was procured from B Braun (Prague, Czech Republic). Deionised ultrapure water was produced inhouse (Simplicity 185 Millipore system, Cambridge, USA). C18 SepPak cartridges from Waters (Milford, USA) were used for post-purification. The *Mycobacterium bovis*-Bacille Calmette-Guérin (BCG) agent and Staphylococcus aureus 25,923 (SA) were donated by the Microbiology Department at Charles University Prague. Any other reagents were purchased from Merck (Darmstadt, Germany) or Fluka (Steinheim, Germany) with the highest purity available. Ultrapure water (Aquatec Water Systems, Inc., CA, USA) was used for all procedures. Suitable doses of [^18^F]FDG were provided in house.

### Radiolabelling and quality control of DOTA-Puromycin

The gallium-68 activity (eluted in 0.1 M hydrochloric acid) was pre-concentrated and purified as previously described^24^. A previously published method was used to prepare [^68^Ga]Ga-DOTA-puromycin^12^. Briefly, DOTA-Puromycin (1.11 nmol/µl) was incubated in gallium-68 (pH 3.5) at a concentration of 0.14 µM at 110 °C for 20 min. The crude product was purified from free gallium-68 and colloidal gallium-68 using a reverse phase C-18 Sep-Pak cartridge. Product was eluted from the cartridge with 300 µl ethanol which was then evaporated (heated at 95 °C for 5 min), and the final product dissolved in 0.9% sterile saline. Radiolabelling efficiency and radiochemical purity were determined by ITLC as validated and published before^12,15^. Also, a system with ITLC-SG plates as the stationary phase and n-propanol/NH_4_OH/H_2_O (55:35:10) as the mobile phase was used. Radio-TLC analysis shows free gallium-68 retaining at the origin (Rf = 0.05) whilst [^68^Ga]Ga-DOTA-puromycin migrates with the solvent front (Rf = 0.85–1.00). Quantification of ITLC-SG plates was performed using a Cyclone Plus Storage Phosphor System (Perkin Elmer, USA). Example iTLC chromatograms and methods are included in Supplementary Figure S2.

### Ethical consideration and containment

All applicable institutional and/or national guidelines for the care and use of animals were followed. The experimental design (Fig. 4) was approved by the committee on animal experimentation of Semmelweis University. All procedures were conducted in accordance with the ARRIVE guidelines as prescribed by the European Communities Council Directive (86/609 EEC). For handling of BCG (contains *Mycobacterium bovis*) biosafety level 2 (BSL-2) practices and procedures for non-aerosol-producing manipulations of clinical specimens were followed. All aerosol-generating activities were conducted in a biosafety cabinet environment.

### Animal study population and design

Severe combined immunodeficient mice (SCID) mice (female, 8 weeks-old, 20.8–23.2 g) were obtained from Toxi-Coop Ltd (Budapest, Hungary) and housed in a controlled animal facility (22 ± 1 °C, 55 ± 10% humidity, 12 h/12 h day-night cycle). The animals had ad libitum access to standard rodent food and purified water. Animals were randomised into two groups (Fig. 4) to assess [^68^Ga]Ga-DOTA-puromycin µPET/MRI sensitivity for imaging mycobacterial infection (Group 1) and address tracer selectivity (Group 2).

Twenty mice (group 1) received a percutaneous (right front leg) BCG dose of 1.5 × 10^5^ colony forming units (CFU) administered in 0.125 ml. A previously described step-by-step protocol was used to administer the same BCG count via direct intrabronchial droplets dissemination (40 μl)^23^. Percutaneous sterile saline injection of 0.125 ml was administered into the left leg as negative control. Another five mice (group 2) received a subcutaneous inoculation of 1.5 × 10^8^ CFU Staphylococcus aureus (SA) into the right armpit area. A 0.125 ml of 1:10 diluted sterile turpentine oil (STO) was injected subcutaneously into the left armpit tissue to induce sterile inflammation. Animals of both groups underwent initial [^68^Ga]Ga-DOTA-puromycin-µPET/MRI imaging 24–48 h after inoculation. Following a 12-week animal monitoring period, animals from group 1 underwent [^68^Ga]Ga-DOTA-puromycin-, and [^18^F]FDG-µPET/MRI imaging on consecutive days.

### Small animal imaging procedures

Animals were immobilised using a MultiCellTM Imaging Chamber (Mediso Ltd., Hungary) with the anaesthesia performed using an in-house protocol^25^. [^68^Ga]Ga-DOTA-puromycin- and [^18^F]FDG imaging were performed on the nanoScan µPET/MRI sequential imaging system (Mediso Ltd., Budapest, Hungary). Prior to [^18^F]FDG imaging animals were prepared as described previously^25^. This protocol includes an overnight fasting period and blood glucose confirmation. Anaesthesia is induced before [^18^F]FDG injection and image acquisition was performed at 30 min post injection for 20 min. MRI acquisition was done for 50 min. The MRI and PET images are automatically co-registered by the acquisition software. No animal preparation was required prior to µPET image acquisition of [^68^ Ga]Ga-DOTA-puromycin (45 min post injection, 20 min scan). Normal list mode with packet time stamping and 50% axial overlap was selected as acquisition parameters. Monte Carlo based scatter modelling was followed by 3-D ordered subsets expectation maximisation reconstruction (4 iterations, 3 subsets, a 400–600 keV energy window, coincidence mode 1:5, voxel size 0.62 mm), detector normalisation and attenuation correction. MRI parameters were set according to previously published protocols^26^. Briefly, a 1 Tesla MRI system was aligned with a dedicated transmit/receive mouse coil. A T2-weighted fast-spin echo was acquired (410 μm in-plane resolution, 0.41 mm slice-thickness, repetition, and echo times of 25/2 ms, field of view (FOV) was 51.2 mm). Four different sequences were used for imaging, two of them for relaxivity measurements and two for basic imaging exploited different contrast mechanisms.

### Image analysis

The reconstructed, re-oriented and co-registered images were further analysed using dedicated image analysis software (InterView Fusion, Mediso Ltd., Hungary; VivoQuant inviCRO LLC, USA) by placing appropriate organ (tissue) volume-of-interests (VOI). These VOIs were delineated manually using MRI images as anatomical reference. Following chronic BCG infection lung tissue of four lung segments (left, superior, middle-posterior, and inferior) were recorded as separate VOIs. The [^18^F]FDG and [^68^ Ga]Ga-DOTA-puromycin average normalised uptake values (NUV) were calculated by dividing the average radioactivity concentration (kBq/mm^3^) per VOI by injected dose per body weight (MBq/g); this data was used to further calculate ratios for target-to-nontarget tissue (T/NT); non-targeted tissue chosen as following: left armpit (e.g., negative control area injected with sterile saline solution BCG pcn., SA, STO) and the intra-scapulary neck/ scruff area (for BCG pulm.) as well as target-to-background ratios(T/Bg; Bg = left, top image areal)), respectively. In addition, NUVs from the following healthy tissue equivalents (cubic voxel volumes) were determined to express target-to-reference ratios (T/Ref): average signal from muscle tissue of both hind legs (for liver, myocardium, spleen, SA infection and STO injection), pleural space and partial pleura (for BCG pulm.) and contralateral percutaneous tissues areal (for BCG pcn.).

### Histology

At their respective endpoint the animals were euthanized by isoflurane overdose and relevant tissues were dissected, fixed in 10% buffered formalin, and embedded in paraffin and sliced (1 μm) for histological processing. Haematoxylin–Eosin (HE; micro-anatomy), Gram (SA) and Ziehl–Neelsen staining (ZN; detecting acid-fast bacilli AFB) were performed as described elsewhere^27–29^. Histological assessments were performed by two experienced pathologists; pathologic tissue changes and the potential bacterial load was examined for lung, myocardium, liver, spleen, and injection sites, i.e., areas of infection/ inflammation. Following chronic BCG infection lung tissue of four lung segments (left, superior, middle-posterior, and inferior) was explored. Such lung segments from 4 animals underwent randomised microscope inspection (n = 10 field of views per tissue). The level of inflammation was graded on a relative scale of 1–4 as following: grade 1—absence of inflammation; grade 2—extent of granuloma or higher number of macrophages/granulocytes; grade 3—onset of necrosis; grade 4—extended necrosis. Deriving from ZN staining, the extent of rod-shaped AFB (*M. bovis*) tissue manifestation was graded on a relative scale of 0–10: grade 0—absence of AFC to grade 10—maximal tissue density of AFC. An example of histology slides is provided as Supplementary Fig. 5.

### Statistical analysis

Statistical analysis was performed with GraphPad Prism Version 8.0 (GraphPad Software, CA, USA). If not mentioned otherwise, the organs or tissues activity concentration was expressed as mean and standard deviation (SD). The significance of two mean values was calculated by one- or two-tailed paired and unpaired Student’s- t-test with the levels of significance (*p*) establishing at *p* < 0.05 (*), < 0.01 (**) and < 0.001 (***). Spearman rank order correlation test was applied to analyse the relationship between NUV and Ziehl–Neelsen staining.

### Supplementary Information

Supplementary Information.

## Data Availability

The datasets generated during and/or analysed during the current study are available from the corresponding author on reasonable request.
