# Supplementary material for: Visualisation of in vivo protein synthesis during mycobacterial infection through [68Ga]Ga-DOTA-puromycin µPET/MRI
Source: Sci Rep. 2024 Aug 20;14:19250. doi: 10.1038/s41598-024-70200-4 (PMC11335739; doi:10.1038/s41598-024-70200-4)
Supplement: Supplementary file 1 — Supplementary Information. [file 41598_2024_70200_MOESM1_ESM.docx]

# Supplementary Figure to: *Visualisation of in vivo protein synthesis during mycobacterial infection through [^68^Ga]Ga-DOTA-puromycin µPET/MRI*

Sebastian Eigner ^1,2^, Janke Kleynhans^3^, Denis R. Beckford Vera^1^, Mike M. Sathekge ^3,4^, Katerina Eigner Henke^2,5^ , and Thomas Ebenhan^3,6#^

*^1^Department of Radiopharmaceuticals, Nuclear Physics Institute, Academy of Science of the Czech Republic, Rez near Prague, Czech Republic ^2^Department of Radiopharmacy, Charles University Prague, Czech Republic;
^3^Nuclear Medicine Research Infrastructure NPC, Pretoria, South Africa,
^4^Department of Nuclear Medicine and Steve Biko Academic Hospital, University of Pretoria, Pretoria, South Africa*

*^5^ Clinic for Nuclear Medicine, University Hospital RWTH Aachen, Aachen, Germany;
^6^Department of Nuclear Medicine University of Pretoria, Pretoria, South Africa;*

**SUPPLEMENT FIGURE S1.** Correlation plot for NUV values from image guided analysis for 68Ga-Pur *versus* 18F-FDG (y=7.51x; N=16). The linear regression (r^2^) analysis returned a value of 0.651.

**
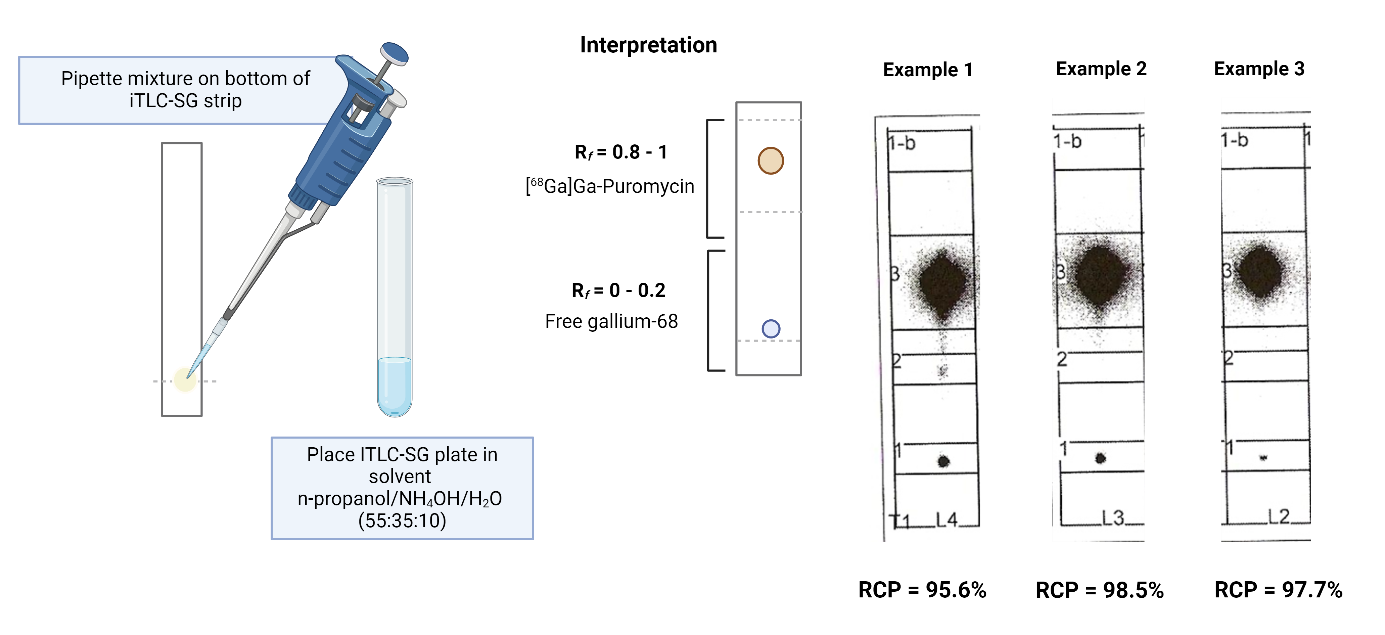
**

**Supplementary Figure S2:** Autoradiography images of 3 synthesis of [68Ga]Ga-DOTA-Puromycin. Radio-TLC analysis shows free gallium-68 retaining at the origin (Rf = 0.05) whilst [^68^Ga]Ga-DOTA-puromycin migrates with the solvent front (Rf =0.85 – 1.00). ITLC was cross-validated with HPLC during previous work – as described in Eigner et al., (2013).

**Supplementary Figure S3**: Ex-vivo biodistribution studies on [^68^Ga]Ga-DOTA-puromycin performed previously in xenograft (PC3 cells) animal models. Tumour data is not included. These measurements are done in animals euthanized 2 hours post-injection and there are 4 animals in the test group.

**
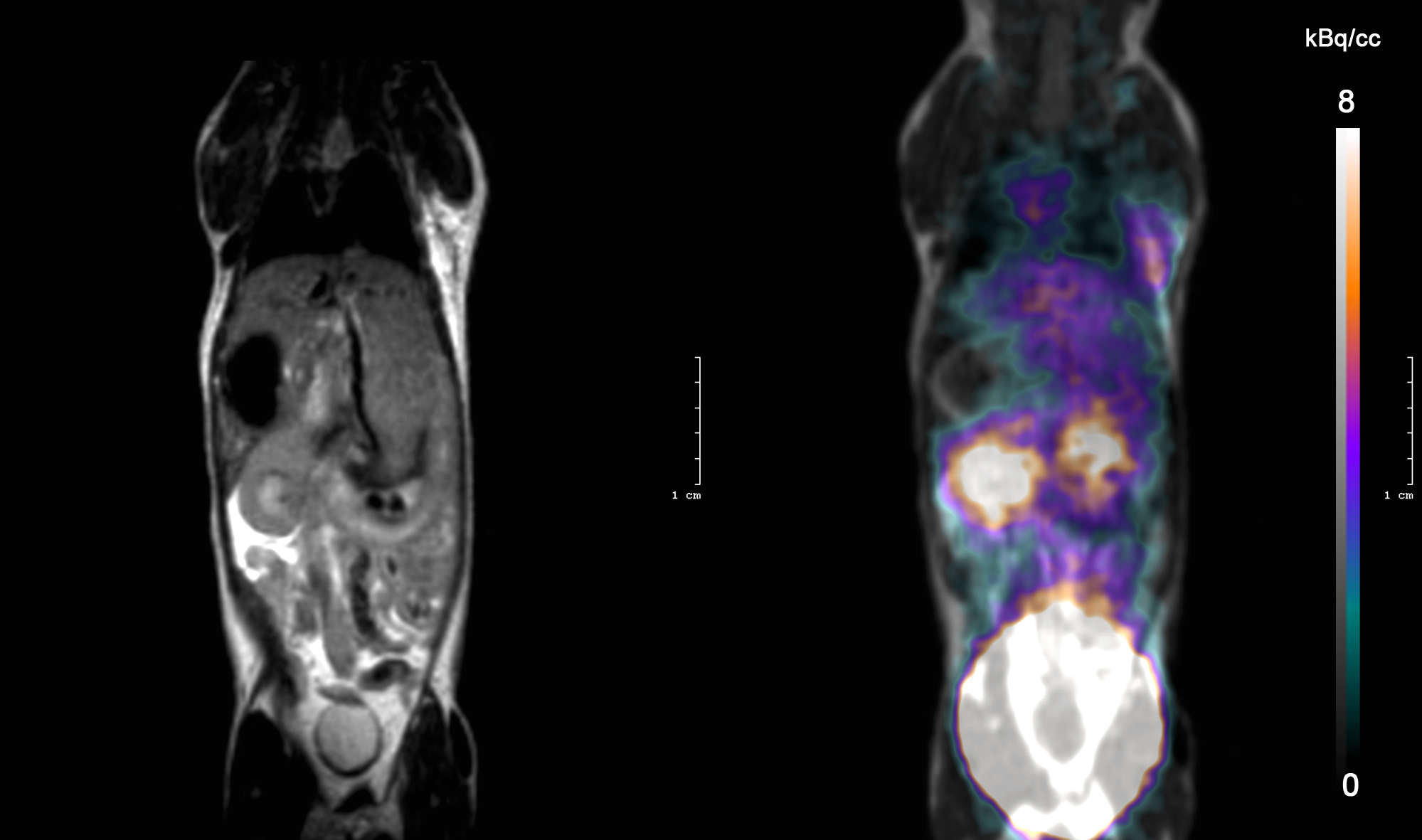
**

**Supplementary Figure S4:** Whole-body [^68^Ga]Ga-DOTA-puromycin-µPET/MRI (representative posterior, coronal whole-body image slice), the full scanner field of view of 6.7 cm including MRI (left) and fused PET/MRI (right) projection from neck – pelvis. Unwanted high bladder activity (bottom right) is limiting the image quality.

| 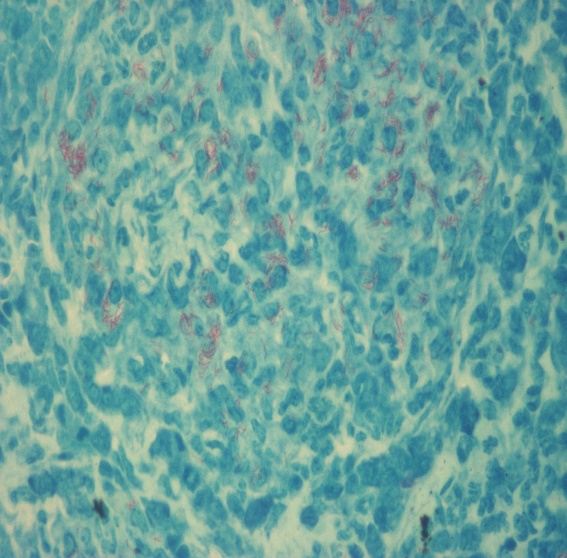 |
| --- |
| +3 Ziehl Neelsen grading |
| 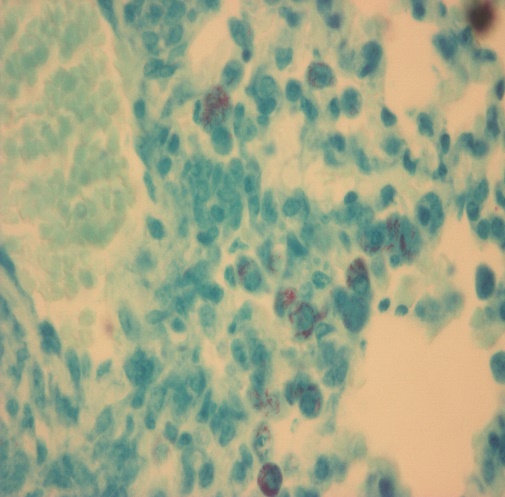 |
| +1 Ziehl Neelsen grading |

**Supplementary Figures S5:** Representative images of the Ziehl Neelsen staining
